# T Cell Costimulation by CD6 Is Dependent on Bivalent Binding of a GADS/SLP-76 Complex

**DOI:** 10.1128/MCB.00071-17

**Published:** 2017-05-16

**Authors:** Johannes Breuning, Marion H. Brown

**Affiliations:** Sir William Dunn School of Pathology, Oxford, United Kingdom

**Keywords:** CD6, GADS, SLP-76, T cells, signal transduction

## Abstract

The cell surface receptor CD6 regulates T cell activation in both activating and inhibitory manners. The adaptor protein SLP-76 is recruited to the phosphorylated CD6 cytoplasmic Y662 residue during T cell activation, providing an activating signal to T cells. In this study, a biochemical approach identified the SH2 domain-containing adaptor protein GADS as the dominant interaction partner for the CD6 cytoplasmic Y629 residue. Functional experiments in human Jurkat and primary T cells showed that both mutations Y629F and Y662F abolished costimulation by CD6. In addition, a restraint on T cell activation by CD6 was revealed in primary T cells expressing CD6 mutated at Y629 and Y662. These data are consistent with a model in which bivalent recruitment of a GADS/SLP-76 complex is required for costimulation by CD6.

## INTRODUCTION

CD6 is a leukocyte surface receptor that regulates T cell activation. As potential targets for therapies that enhance or inhibit immune responses, it is imperative to understand how these receptors regulate T cell activation. CD6 function is regulated by interaction with a cell surface ligand, CD166, also known as activation-induced leukocyte adhesion molecule (ALCAM). CD6 has roles in both restraining and promoting T cell activation (1–4). Despite recent advances in fundamental understanding with molecular characterization of the extracellular and intracellular regions of CD6 and the availability of CD6-deficient mice, the molecular basis of these dual effects is not well characterized (3, 5, 6).

CD6 has the longest cytoplasmic region, 244 amino acids, without catalytic function in the immune system (7). Costimulation by CD6 depends on ligand binding at the cell surface and an interaction between the phosphorylated C-terminal tyrosine Y662 and the adaptor protein SH2 domain-containing leukocyte molecule of 76 kDa (SLP-76) (1, 4, 8, 9). The interaction between CD6 and SLP-76 is clearly important for costimulation by CD6, but there are data to indicate that other regions of the extensive cytoplasmic region regulate its function through serine and tyrosine phosphorylation (10–12).

We identify a novel interaction between Y629 in the cytoplasmic region of CD6 and the SLP-76 binding partner GRB2-related adaptor downstream of Shc (GADS). The biochemical data provided evidence that the GADS/SLP-76 complex bound bivalently to the CD6 C terminus. Mutation of both the GADS and SLP-76 binding sites completely abrogated CD6-mediated costimulation in the Jurkat cell line and primary CD4^+^ T cells, suggesting that cooperative binding between GADS and SLP-76 is essential for CD6 to activate signaling. Removal of the interactions required for costimulation revealed the capacity of CD6 to mediate inhibitory signaling.

## RESULTS

### CD6 Y629 isolated GADS, GRB2, and TSAd from T cell lysates.

To understand the molecular basis of signal transduction by CD6, we have taken the approach of identifying potential binding partners for phosphorylated tyrosine-based motifs using peptides and cell lysates, characterizing the interactions biochemically, and assessing the functional consequences in cellular assays. This approach was successful in identifying the interaction between the C-terminal tyrosine residue Y662 and SLP-76 and its importance for costimulation by CD6 (1). In this study, we focus on investigating the role of Y629 based on evidence that its phosphorylation, similarly to Y662, is regulated by T cell activation and that it is contained within a motif predicted to bind an SH2 domain (11, 13).

A peptide containing the phosphorylated CD6 Y629 residue immobilized in a multivalent form on magnetic beads was used to isolate proteins from lysates of the Jurkat human T cell line. Three proteins were isolated: GADS, growth factor receptor-bound protein 2 (GRB2), and T cell-specific adaptor protein (TSAd), otherwise known as SH2D2A (Table 1). These proteins were the only proteins exclusively present in the specific pulldown and not in the control in at least two experiments. These data confirm the prediction that the Y629 residue of CD6, which is contained within a YXN motif, would preferentially bind the SH2 domains of these three adaptor proteins (11, 13).

**TABLE 1:** A CD6 pY629 peptide isolated SH2 domain-containing adaptor proteins from Jurkat T cell lysates^*a*^

Protein	No. of unique peptides (no. of independent expts)^*b*^	% sequence coverage^*c*^
GADS	21–43 (3)	60–81
GRB2	18–40 (3)	59–79
TSAd	3–4 (2)	11–14

a Proteins isolated from Jurkat T cell lysates by multivalent CD6 pY629 were identified by mass spectrometry.

b Peptides found only in the target protein.

c Protein sequence covered by the identified peptides.

### CD6 Y629 binds directly to the SH2 domains of GADS, GRB2, and TSAd.

**FIG 1:**
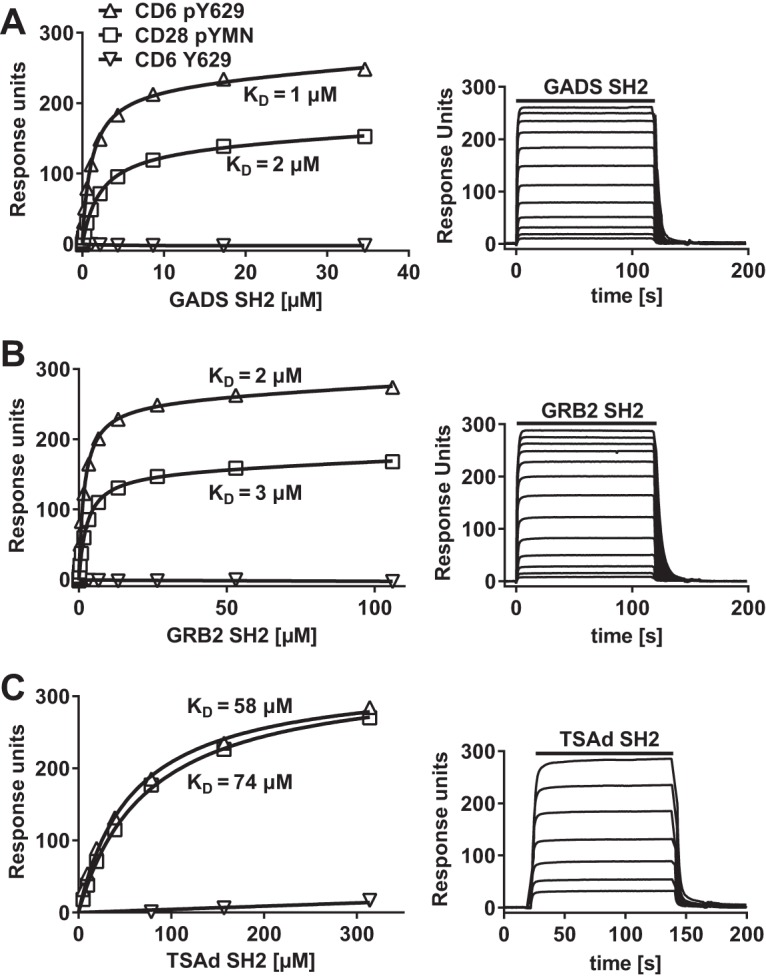
CD6 Y629 binds directly to the SH2 domains of GADS, GRB2, and TSAd. Equilibrium binding fitted curves and *K_D_*s (left) were derived from the sensorgram data (right) for 2-fold serial dilutions of GADS (35 μM) (A), GRB2 (106 μM) (B), and TSAd (314 μM) (C) SH2 domains over immobilized peptides. Background signals for proteins over streptavidin were subtracted.

Isolation from cell lysates gives an indication of likely binding partners but does not distinguish between direct and indirect interactions that may be a consequence of an association with large complexes. We measured the direct binding of purified monomeric SH2 domains to the phosphorylated CD6 Y629 peptide at physiological temperature using surface plasmon resonance (SPR) (Fig. 1). The sensorgram traces for all three proteins binding to phosphorylated CD6 Y629 are consistent with monomeric binding of all three proteins (Fig. 1, right). Equilibrium binding data were plotted to determine dissociation constants (*K_D_*s) (Fig. 1, left). GADS and GRB2 bound with similar *K_D_*s to phosphorylated Y629, at 1 to 3 μM and 2 to 4 μM, respectively (Fig. 1A and B and Table 2). As an indication that the recombinant proteins were active, the SH2 domains of GADS and GRB2 bound to a phosphorylated CD28-derived peptide containing the YXN motif with an affinity (*K_D_* = 2 to 3 μM) similar to that determined by previous measurements (Fig. 1 and Table 2) (14). Compared with GADS and GRB2, TSAd bound to phosphorylated CD6 Y629 and CD28 peptides with an affinity that was more than an order of magnitude lower (Fig. 1C and Table 2).

**TABLE 2:** SH2 domain-containing adaptor proteins bind directly to a CD6 pY629 peptide^*a*^

Peptide	*K_D_* (μM)		
	GADS SH2	GRB2 SH2	TSAd SH2
CD6 pY629	1–3	2–4	26–58
CD28 pYMN	2–3	3	74^*b*^

a The *K_D_* was determined from equilibrium binding of soluble recombinant SH2 domains to the immobilized peptide at 37°C by using SPR (*n* = 3).

b *n* = 1.

### The GADS/SLP-76 complex is recruited to CD6 Y629 and Y662.

**FIG 2:**
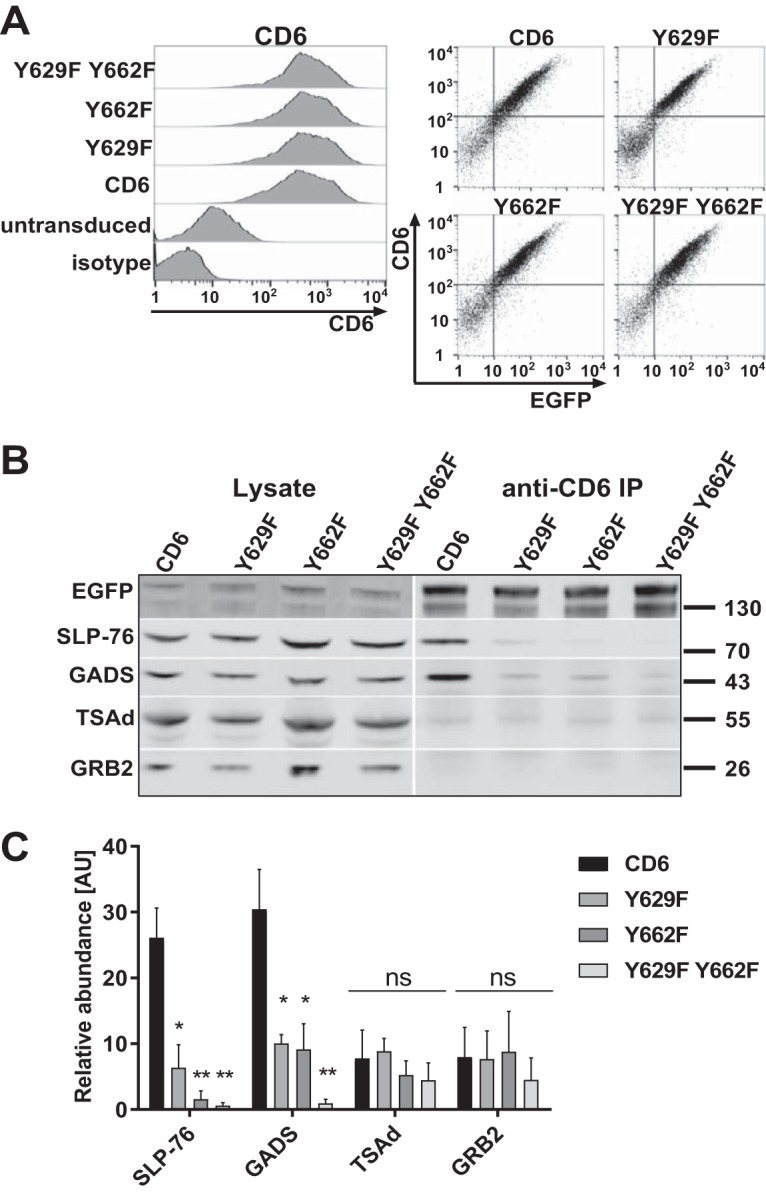
The GADS/SLP-76 complex is recruited to CD6 Y629 and Y662. (A, left) Human CD6, Y629F and Y662F single mutant, and Y629F Y662F double mutant proteins fused to EGFP and stained with a CD6 MAb (T12.1) were expressed at similar levels on Jurkat cells. (Right) CD6 surface staining is correlated with the EGFP signal. (B and C) CD6 was immunoprecipitated from Jurkat cells (3 × 10^6^ cells per sample). Western blots of lysates and CD6 immunoprecipitates (IP) were probed for SLP-76, GADS, TSAd, GRB2, and EGFP to detect the CD6-EGFP fusion protein. A representative blot (B) and combined data from densitometric analyses for three experiments (C) are shown. The bars (means ± standard errors of the means) represent the ratios of coimmunoprecipitated CD6/CD6 in the lysate normalized to the ratio of immunoprecipitated CD6/CD6 in the lysate to measure the relative abundance, in arbitrary units (AU), of intracellular proteins in CD6 immunoprecipitates. The unpaired Student *t* test was used to compare values for the mutants with those for CD6. *, *P* < 0.05; **, *P* < 0.01; ns, not significant.

We tested for the association of CD6 with the three adaptor proteins GADS, GRB2, and TSAd in cells using a Jurkat T cell line transduced with CD6, the Y629F or Y662F single mutant, or the Y629F Y662F double mutant fused to enhanced green fluorescent protein (EGFP) (Fig. 2A). Endogenous CD6 in Jurkat cells was expressed at a low level and was unlikely to obscure the effects of the more highly expressed transduced CD6 (Fig. 2A). In flow cytometry analyses, CD6 monoclonal antibody (MAb) and EGFP were correlated, showing that the fusion proteins were expressed at the surface at similar levels in each of the cell lines, which justified quantifying CD6 levels by using an EGFP antibody in Western blot analyses (Fig. 2B). Cells were treated with pervanadate to maximize the levels of phosphorylated CD6 and lysed, and CD6 was immunoprecipitated by using a CD6 MAb (MEM-98) and examined for associated proteins by Western blotting (Fig. 2B and C).

Consistent with flow cytometry data, lysates from the different cell lines contained similar levels of CD6 as detected by Western blotting for EGFP expression (Fig. 2B). The two bands for CD6 that were observed previously most likely represent differently glycosylated forms of CD6 (12). SLP-76, GADS, GRB2, and TSAd were detected in the lysates of each cell line (Fig. 2B, left). The adaptor proteins differed in the relative amounts associated with immunoprecipitated CD6 (Fig. 2B, right). These data were quantified (Fig. 2C). SLP-76 coimmunoprecipitated with CD6, showing that the C-terminal fusion of EGFP with CD6 does not hinder the association of CD6 with intracellular binding partners (1). Of the three candidates for binding to the Y629 YXN motif in CD6, GADS, GRB2, and TSAd, only GADS was significantly enriched in the wild-type CD6 immunoprecipitates, indicating that it is the main interaction partner (Fig. 2B, right, and C). Coprecipitation of SLP-76 and GADS with CD6 depended on phosphorylation and was not observed in the absence of pervanadate treatment (data not shown).

Mutation of Y629 or Y662 resulted in a reduced association of both SLP-76 and GADS with CD6. Mutation of both tyrosine residues Y629 and Y662 prevented binding entirely (Fig. 2B, right, and C). Mutation of these residues had no effect on the amounts of GRB2 and TSAd detected in CD6 immunoprecipitates, providing further evidence for GADS and SLP-76 being specific intracellular ligands for CD6 (Fig. 2B, right, and C).

**FIG 3:**
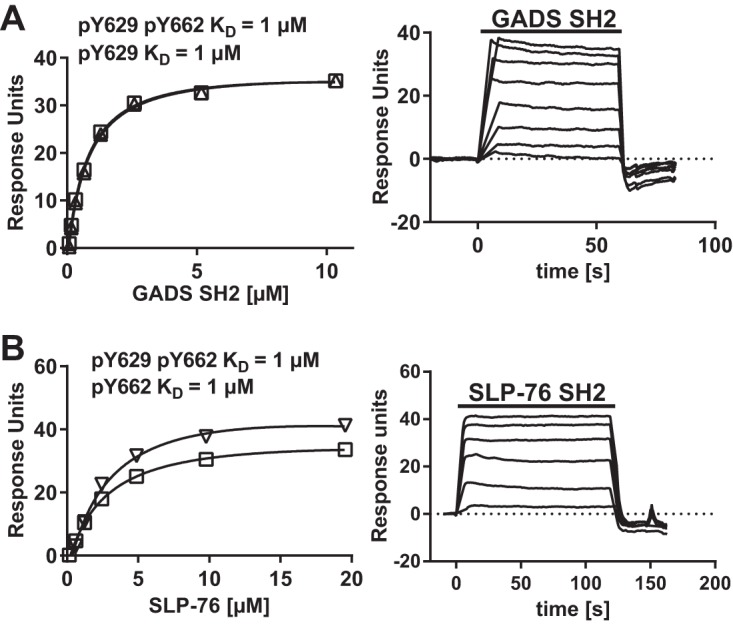
GADS SH2 and SLP-76 SH2 are specific for CD6 Y629 and Y662, respectively. Equilibrium binding fitted curves and *K_D_*s (left) were derived from SPR sensorgram data (right) for 2-fold serial dilutions of GADS (10 μM) (A) and SLP-76 (20 μM) (B) SH2 domains over the long CD6 peptides. There was no binding of the GADS SH2 domain to pY662 or of the SLP-76 SH2 domain to pY629 (data not shown). Background signals for proteins over streptavidin were subtracted.

SPR analysis with long peptides containing both Y629 and Y662 phosphorylated at either or both tyrosine residues confirmed the specificity of the SH2 domains of GADS and SLP-76 for the CD6 Y629 and Y662 motifs, respectively (Fig. 3 and Table 3). The data suggest a model in which GADS and SLP-76 bind cooperatively to CD6. Coprecipitation of GADS with CD6 was not observed in Jurkat (J14) cells, which lack SLP-76 (data not shown). GADS and SLP-76 interact with each other independently of their SH2 domains. A GADS SH3 domain binds to a proline-rich region of SLP-76 (15). This configuration means that both SH2 domains are available for bivalent binding to two phosphorylated tyrosine residues in close proximity. CD6 Y629 is the closest tyrosine residue to CD6 Y662 in the linear amino acid sequence of the cytoplasmic region.

**TABLE 3:** SLP-76 and GADS bind specifically to CD6 Y629 and Y662, respectively^*a*^

CD6 peptide	*K_D_* (μM) (range)	
	GADS SH2	SLP-76 SH2
pY629	0.2–0.6	
pY662		0.3–2
pY629 pY662	0.1–2	0.3–2

a *K_D_*s were determined from equilibrium binding of soluble recombinant SH2 domains to immobilized long CD6 peptides at 37°C using SPR (Fig. 3) (*n* = 3).

### Costimulation by CD6 is dependent on CD6 Y629 and Y662 in a Jurkat T cell line.

**FIG 4:**
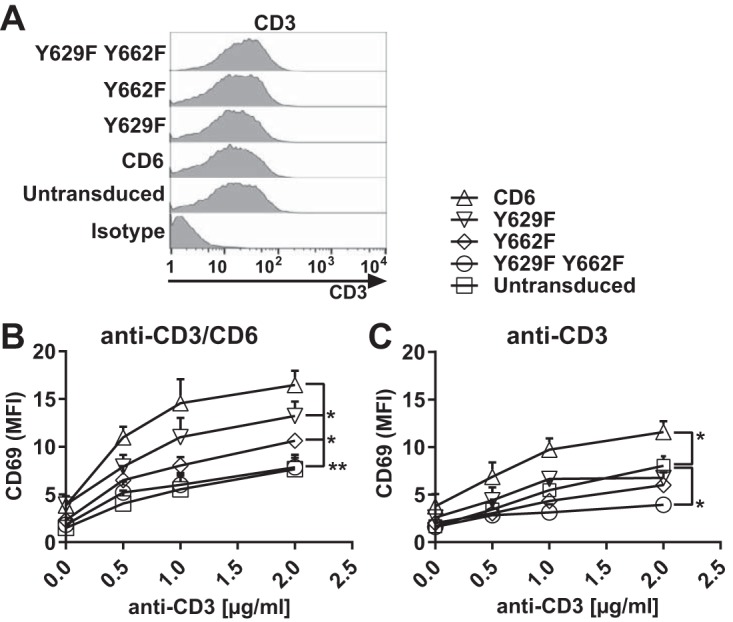
Costimulation by CD6 is dependent on CD6 Y629 and Y662 in Jurkat cells. Flow cytometry analysis of Jurkat cells transduced with human CD6, the Y629F or Y662F single mutant, or the Y629F Y662F double mutant fused with EGFP and stained with CD3 MAb UCHT1, which showed that CD3 levels were unchanged by transduction (A), or stimulated with different concentrations of CD3 MAb with (B) or without (C) CD6 MAb (T12.1) (2.5 μg/ml) and measured for CD69 MFI on EGFP-positive cells. Compared with untransduced cells, the CD69 MFI was increased in cells transduced with CD6, but the effect of CD6 was reduced by the Y629F or Y662F single mutation (B) or the Y629F Y662F double mutation (B and C). *, *P* < 0.05; **, *P* < 0.01. Combined data from three experiments (means ± standard errors of the means) are shown. The unpaired Student *t* test was used to compare the maximum effect (*E*_max_) values of each dose-response curve.

We compared the effects of Y629 and Y662 on costimulation in a Jurkat T cell line transduced with CD6, the Y629F or Y662F single mutant, or the Y629F Y662F double mutant as EGFP fusion proteins (Fig. 2). As was observed previously, transduction did not affect CD3 expression, and the transduced Jurkat T cells were expected to respond equally to T cell receptor (TCR)-mediated stimulation using CD3 MAbs (1, 2) (Fig. 4A). Jurkat T cells were stimulated with immobilized CD3 and/or CD6 (T12.1) MAbs to mimic ligand engagement, and the enhancement of the levels of the cell surface activation marker CD69 was measured by flow cytometry as an indication of T cell activation. The mean fluorescence intensity (MFI) of CD69 was measured on cells gated for transduced CD6 as monitored by EGFP expression.

Cells transduced with CD6 and stimulated with both CD3 and CD6 MAbs showed an enhancement of CD69 levels compared with those in untransduced cells (Fig. 4B). Mutation of Y662 or Y629 reduced CD6-mediated signaling, with the SLP-76 binding site having the dominant effect (Fig. 4B). Mutation of both Y629 and Y662 brought the signal down to the level of that in untransduced cells (Fig. 4B). These results show that costimulation by CD6 in Jurkat cells is dependent on the GADS binding site Y629 and the SLP-76 binding site Y662, and mutation of both residues abolished CD6-mediated costimulation. The functional and biochemical data support the interpretation that there is cooperative binding by GADS and SLP-76 to phosphorylated CD6 Y629 and Y662.

The CD6-mediated costimulatory effects were dependent on the extracellular engagement of CD6. In response to CD3 MAb stimulation alone, only a low level of CD69 upregulation by Jurkat cells expressing CD6 was observed (Fig. 4C). Notably, in the absence of CD6 MAb cross-linking, an inhibitory effect of the Y629F Y662F double mutant was observed (Fig. 4C).

### Costimulation by CD6 is dependent on CD6 Y629 and Y662 in primary CD4^+^ T cells.

We tested the importance of Y629 and Y662 for the costimulatory effects of CD6 in human primary T cells. Having established that the functional effects of CD6 on proximal events in TCR-mediated signal transduction can be studied by using the transduced EGFP fusion proteins and a CD6 MAb to mimic ligand engagement, we introduced a further modification to facilitate analysis in primary human T cells. The human CD6 MAb T12.1 used for cross-linking of CD6 in Jurkat cells binds the N-terminal domain of CD6 (16; L. I. Garner and M. H. Brown, unpublished data). The rat CD6 MAb OX52 was also likely to bind domain 1, as it recognizes CD6 lacking domain 3 (17; M. H. Brown, unpublished data). We swapped domain 1 and part of domain 2 of the extracellular region of human CD6 for the rat counterpart. We confirmed that chimeric CD6 was expressed and responded to cross-linking with the rat CD6 MAb (data not shown).

**FIG 5:**
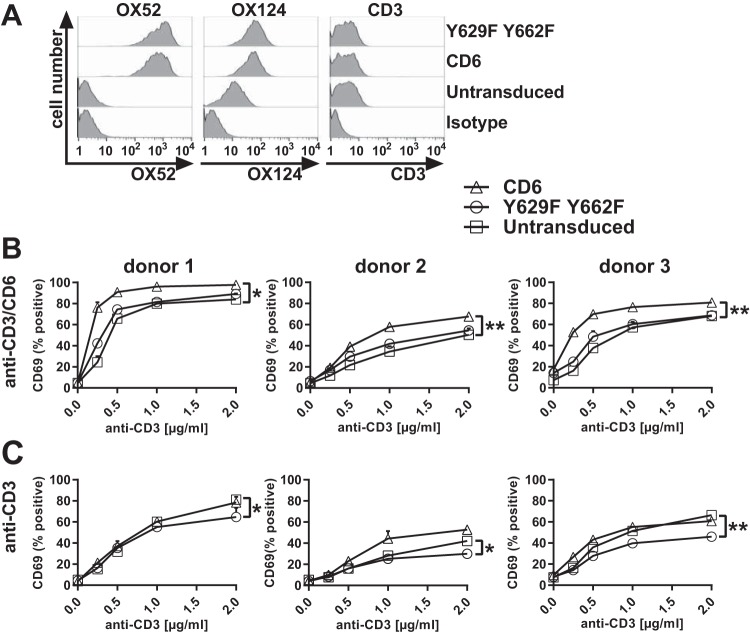
Costimulation by CD6 is dependent on CD6 Y629 and Y662 in primary CD4^+^ T cells. Flow cytometry analysis of primary CD4^+^ T cells transduced with rat domain 1-containing human CD6 or the Y629F Y662F double mutant protein fused to EGFP. (A) Cells stained with a rat domain 1-containing CD6 MAb (OX52) (left), a human domain 3-containing CD6 MAb (OX124) (middle), and a CD3 MAb (UCHT1) (right) show that there are similar levels of CD6 expression on transduced T cell blasts with ∼40-fold-higher MFIs and that CD3 levels in T cells were unchanged compared with those in untransduced cells. Negative isotype controls are shown for each MAb. (B and C) Cells were stimulated with different concentrations of CD3 MAb with (B) and without (C) CD6 MAb (OX52) (5 μg/ml) and measured for the percentage of CD69^+^ EGFP-positive cells. Compared with those for untransduced cells, the percentages of CD69^+^ cells were increased in cells transduced with CD6, but the effect of CD6 was reduced by the Y629F Y662F double mutation. *, *P* < 0.05; **, *P* < 0.01. Combined data from three experiments (means ± standard errors of the means) are shown. The unpaired Student *t* test was used to compare the *E*_max_ values of each dose-response curve.

Primary CD4^+^ T cell blasts were transduced with rat CD6 domain 1 followed by human CD6 containing the wild-type or the Y629F Y662F double mutant cytoplasmic region fused to EGFP. Chimeric CD6 and the cytoplasmic mutant were expressed on the surface of T cells, with the rat CD6 MAb OX52 giving a high signal for transduced CD6 and not binding to untransduced cells (Fig. 5A). The levels of transduced CD6 were estimated to be 40 to 50 times higher than the level of endogenous CD6 by staining with a CD6 domain 3 MAb (OX124) that recognizes both transduced and endogenous CD6 (Fig. 5A) (1). Levels of CD3 were unchanged by the transduction of CD6 (Fig. 5A).

Cells were stimulated with immobilized CD3 and rat CD6 MAbs to measure the effect of CD6 on the expression of CD69. Cells were gated for EGFP expression, as it correlated with rat CD6 MAb binding (data not shown). Up to 50% of the T cells expressed transduced CD6 (data not shown). In T cell blasts, the percentage of CD69-positive cells but not the MFI of CD69 expression increased significantly in response to stimulation. Stimulation of T cell blasts transduced with CD6 containing an intact cytoplasmic region with CD3 MAb and rat CD6 MAb increased the percentage of CD69-positive cells compared to that for untransduced cells from three donors (Fig. 5B). Similarly to Jurkat cells, responses by cells expressing CD6 with mutated Y629 and Y662 gave activation levels comparable to those of untransduced T cells for all three donors (Fig. 5B). These data support the conclusions drawn from analyses of Jurkat T cells showing that CD6 Y629 and Y662 are necessary for CD6-mediated costimulation. Costimulation by CD6 was dependent on cross-linking with a CD6 MAb, consistent with physiological regulation by ligand engagement of the extracellular region (Fig. 5B and C) (1).

Similarly to the Jurkat cells, the expression of CD6 without cross-linking by a CD6 MAb revealed an inhibitory effect in all three donors in response to stimulation with CD3 MAb (Fig. 4C). The inhibitory effect, as in the Jurkat cells, was revealed only in cells expressing CD6 with mutated Y629 and Y662 (Fig. 4B and 5C).

**FIG 6:**
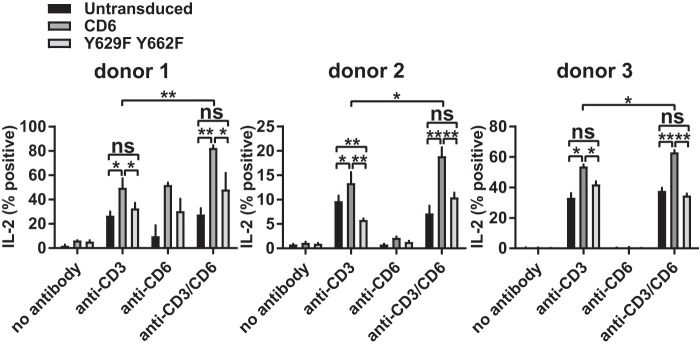
CD6-mediated IL-2 production is dependent on CD6 Y629 and Y662 in primary CD4^+^ T cells. T cell blasts transduced with rat domain 1-containing human CD6 and the Y629F Y662F mutant fused to EGFP were stimulated with CD3 MAb (2 μg/ml) and/or CD6 MAb (OX52) (5 μg/ml), and the percentage of IL-2-positive, EGFP-positive cells was measured by flow cytometry. Combined data from three experiments (means ± standard errors of the means) are shown. The unpaired Student *t* test was used to compare values (*, *P* < 0.05; **, *P* < 0.01).

In addition to the effects on more proximal events in T cell activation, including CD69 expression and phosphorylation, CD6 modifies more downstream functions (1, 4). Our previous study showed that interleukin-2 (IL-2) production was reduced by the single point mutation Y662F in a cell line (1, 4). We measured the effect of CD6 containing the double Y629F Y662F mutation on IL-2 production in human T cell blasts expressing the transduced chimeric CD6-EGFP fusion proteins by flow cytometry. Similarly to CD69, individual cells did not upregulate the level of cytoplasmic IL-2, but changes in the percentages of IL-2-positive cells in response to stimulation with immobilized MAbs were detected (Fig. 6).

The percentage of IL-2-positive cells was increased in cells expressing transduced CD6 in response to stimulation with both CD3 and rat CD6 MAbs (Fig. 6). Mutation of Y629 and Y662 decreased the percentage of IL-2-positive cells to the level in untransduced cells (Fig. 6). Mutation of Y629F alone partially reduced the percentage of IL-2-positive cells (data not shown). This pattern was also observed in the absence of CD6 cross-linking, with reduced differences between the intact and mutated CD6 cytoplasmic regions and overall IL-2 production in all three donors (Fig. 6). The CD6-specific costimulatory effects in the absence of CD6 cross-linking by a MAb are most likely caused by CD6 binding to its ligand CD166 on T cell blasts. The rat CD6 domain 1 MAb does not interfere with the interaction between CD6, which is mediated by domain 3, and its ligand CD166 by SPR analysis (Brown, unpublished). The inhibitory effect of CD6 in the absence of Y629 and Y662 was also apparent in primary T cells, reaching significance for donor 2 (Fig. 6).

In summary, in addition to the upregulation of CD69 expression, costimulation by CD6 in primary T cells affects IL-2 production, and this is dependent on the CD6 Y629 and Y662 residues and cross-linking CD6.

## DISCUSSION

**FIG 7:**
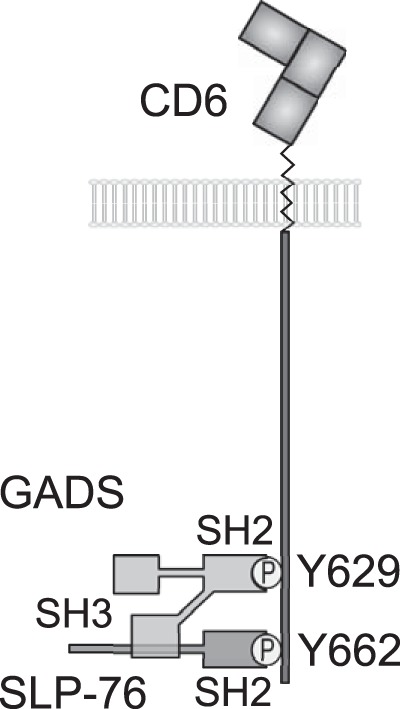
Model for bivalent recruitment of the GADS/SLP-76 complex by CD6. The GADS and SLP-76 SH2 domains bind to the phosphorylated tyrosine residues Y629 and Y662, respectively. The C-terminal SH3 domain of GADS binds to a proline-rich region of SLP-76, and we provide evidence that the GADS/SLP-76 complex binds bivalently to the CD6 C terminus.

We have identified the two most C-terminal tyrosine residues, Y629 and Y662, in the long CD6 cytoplasmic tail as being critical for costimulation by CD6. These residues, Y629 and Y662, when phosphorylated, bind to the SH2 domains of the adaptor proteins GADS and SLP-76, respectively. The data support a model in which a GADS/SLP-76 complex binds bivalently to Y629 and Y662, and this cooperative binding is essential for CD6-mediated costimulation in T cells (Fig. 7). Peptide pulldown experiments and SPR analysis revealed a remarkable specificity of both CD6 Y629 and Y662 for SH2 domains and vice versa: the binding of the SH2 domains of GADS and SLP-76 to CD6 was specific for Y629 and Y662, respectively (Tables 1 and 3 and Fig. 3) (1).

GRB2 and GADS bound equally effectively to CD6 by SPR analysis, and they are present at similar cytosolic concentrations in T cells (18). Although, compared with GADS, GRB2 is enriched in T cell membrane fractions containing signaling proteins associated with the T cell receptor (19), GADS was preferentially associated with CD6. GRB2 and GADS differ in the specificities of their SH3 domains (20). The C-terminal SH3 domain of GADS flanking the SH2 domain binds a proline-rich motif in SLP-76 (15). The affinity of the interaction (*K_D_* = 0.2 μM at 25°C) is 50-fold higher than that for the C-terminal SH3 domain of GRB2 (*K_D_* = 10 μM at 25°C) binding the same region in SLP-76, making it unlikely that a tripartite complex of GRB2, GADS, and SLP-76 would occur, even though the stoichiometry of binding of GADS to SLP-76 and of GRB2 to its ligand SOS can be greater than 1:1 (21, 22). Cooperative binding by a complex of GADS and SLP-76 provides a plausible explanation for the preferential coprecipitation of GADS with CD6 compared with GRB2. The SPR measurements show that the interactions between the phosphorylated tyrosine residues and SH2 domains are transient, with rapid dissociation (1). Bivalent binding by a complex of SLP-76 and GADS explains how the interaction was maintained during washing steps in coimmunoprecipitation experiments. The interaction between a GADS/SLP-76 complex and CD6 is likely to be very specific, as the arrangement of the two C-terminal tyrosine motifs is unique to CD6. The reproducible finding that CD6 recruits SLP-76 and the well-characterized association between GADS and SLP-76 strengthen the conclusion that these two adaptor proteins are the most important binding partners for the C terminus of CD6 (1, 6, 20). Altering concentrations of interacting adaptor proteins and the receptor artificially or in an immune response may allow competition by other contenders (23).

In models of T cell activation through the T cell receptor, GADS and SLP-76 have been best characterized as being recruited to the transmembrane adaptor protein linker for activation of T cells (LAT). The GADS SH2 domain binds to LAT preferentially at YXN motifs at Y171 and Y191 (24). The C-terminal SH3 domain of GADS flanking the SH2 domain binds a proline-rich motif in SLP-76 (15). CD6 has been suggested to be an alternative scaffold protein to LAT (6). The compositions of signaling complexes recruited by CD6 and LAT will differ; for example, when bound to LAT via GADS, the SH2 domain of SLP-76 is available for binding to the adaptor ADAP or the kinase HPK1 in contrast to being occupied when bound to CD6 (25).

The transmembrane proteins CD6 and LAT do not contain enzymatic activity, nor do the adaptors SLP-76 and GADS. LAT has a role in the direct recruitment of enzymes to propagate downstream signaling, binding directly to the SH2 domains of phospholipase C-γ1 (PLC-γ1) and IL-2-inducible T cell kinase (ITK) (25). These enzymes have been implicated in CD6 function, with CD6 affecting Ca^2+^ responses and ITK being detected in CD6 immunoprecipitates (2, 11, 26). It is possible that the GADS/SLP-76 complex bound to CD6 contributes to the recruitment of downstream enzymes such as PLC-γ1 and ITK independently of LAT. Interactions between the SH2 or SH3 domains of enzymes such as PLC-γ1 and ITK and SLP-76 bound to CD6 could be stabilized by an additional interaction with other regions of the CD6 cytoplasmic region (11, 25, 27, 28). T cells from a patient with a premature stop codon in LAT responded to CD3 MAb stimulation with normal Ca^2+^ influx, indicative of recruitment and enzymatic activity of PLC-γ1 (29). Engagement of CD6 by its ligand CD166 may have rescued PLC-γ1 function. The expression of CD6 in a LAT-deficient Jurkat cell line did not substitute for LAT, which may be because the cells did not express CD166 and CD6 was not cross-linked (29).

The costimulatory effects of CD6 through Y629 and Y662 are regulated by the interaction of its extracellular region with CD166, which we have mimicked using CD6 MAbs. The expression of CD6 with low or without any detectable engagement by a cell surface ligand on an apposing cell exerts a restraint on T cell activation (1–3). Our experiments with CD6 lacking the residues necessary for the activating effects of CD6 revealed inhibitory signaling when the extracellular region of CD6 was not cross-linked. These data indicate that activation is dominant over inhibition above a critical level of ligand engagement. A comprehensive understanding of the molecular mechanisms of regulation by CD6 has implications for other receptors that have positive and negative effects on T cell responses, particularly CD5, to which CD6 is related (30).

## MATERIALS AND METHODS

### Peptide pulldown and mass spectrometry.

Jurkat T cells were stimulated with 500 ng/ml ionomycin and 50 ng/ml phorbol myristate acetate (PMA) for 24 h to induce the expression of potential CD6 phosphorylated Y629 (pY629) binding partners. Cells (2 × 10^8^ per sample) were lysed in 1 ml, and lysates were incubated with 250 μl Dynabeads M-280 streptavidin (Life Technologies) coated with 50 μg biotinylated CD6 pY629 peptide (biotin-SSGEWpYQNFQP) or a control peptide (biotin-HVDNEYSQPPRNS) (Peptide Protein Research Ltd., Hampshire, UK) for 18 h at 4°C. The beads were washed three times with 1 ml 0.1% Triton X-100 in phosphate-buffered saline (PBS), with transfer into fresh tubes for each wash, and proteins were eluted with 0.1 M glycine-HCl (pH 2.5) for 1 h at 70°C. For mass spectrometry analysis, eluted proteins were then processed on a Vivacon 500 filter (Vivaproducts) (31). Proteins were denatured with 8 M urea for 1 h, reduced with 10 mM dithiothreitol (DTT) for 1 h, and alkylated with 2 mg/ml iodoacetamide (Sigma-Aldrich) for 1 h at room temperature, followed by digestion with 1 μg trypsin for 20 h at 37°C. Peptides were eluted with 0%, 50%, and 100% acetonitrile in 0.1% formic acid; desalted; and resuspended in 5% acetonitrile–0.1% formic acid. Peptides were injected into the Ultimate 3000 RSLCnano high-performance liquid chromatography system (Dionex)–tandem mass spectrometry Q Exactive Orbitrap machine by using a 25-cm- by 75-μm-inner-diameter picotip analytical column (New Objective) in the central proteomics facilities at the Sir William Dunn School of Pathology, University of Oxford. The resulting spectra were analyzed by using the Mascott search engine (32).

### Expression of recombinant CD6.

A pHR-SIN-BX-IRES-Em (Emerald) lentiviral expression vector (33) was modified by replacement of the XhoI-NotI fragment encoding the internal ribosome entry site (IRES) and Em with EGFP (pHR-EGFP). Human CD6 domain 1 and part of domain 2 were swapped for the rat equivalent (GenBank accession number NM_175577) with the joining site VNCSGAEAYLWD (the amino acids encoded by an internal HindIII site in human CD6 are underlined). The coding sequences for human CD6 (GenBank accession number HSU34623), chimeric rat domain 1-containing human CD6, and cytoplasmic mutants were cloned via a Bluescript shuttle vector as a BamHI-SalI fragment into BamHI-XhoI-cut pHR-EGFP to encode fusion proteins with the joined (underlined) ISAASSMVSK site between the CD6 C terminus and the EGFP N terminus.

### Soluble recombinant proteins and surface plasmon resonance.

Recombinant His-tagged SH2 domains of GADS, GRB2, and TSAd in the popinF vector (provided by Louis Bird, Oxford Protein Production Facility, Harwell, Oxfordshire, UK) and SLP-76 (1) were expressed in Escherichia coli Rosetta(DE3)/pLysS competent cells (Novagen) and purified by nickel affinity chromatography and size exclusion chromatography. Peptides for CD6 pY662 (biotin-PDSTDNDDpYDDISAA), CD6 Y629 (biotin-SSGEWYQNFQP), CD6 pY629 (biotin-SSGEWpYQNFQP), and CD28 pYMNM (biotin-LLHSDpYMNMTP) were immobilized at ∼25 response units on streptavidin-coated CM5 chips in a BIAcore 3000 machine (Peptide Protein Research Ltd., Hampshire, UK) (1, 14). Long CD6 peptides (biotin-STSSGEWYQNFQPPPQPPSEEQFGCPGSPSPQPDSTDNDDpYDDISAA) phosphorylated at Y629 (pY629), Y662 (pY662), or both (pY629 pY662) were immobilized at ∼50 response units. Serially diluted proteins were injected over the chip at 10 μl/min at 37°C. Background signals of proteins over a streptavidin-coated flow cell were subtracted, equilibrium binding data were plotted, and *K_D_*s for 1:1 Langmuir binding were determined.

### Coimmunoprecipitation.

Jurkat T cells (3 × 10^6^ per sample) transduced with CD6, the Y629F or Y662F single mutant, or the Y629F Y662F double mutant fused to EGFP were lysed with 1% Triton X-100 in 100 μl and incubated with CD6 MAb MEM98 (5 μg; Abcam) for 18 h at 4°C. Lysates were then incubated with protein A/G beads (250 μg; Thermo Scientific) for 1 h at room temperature. Beads were washed and eluted with Laemmli buffer at 95°C for 5 min. Cell lysates (8 × 10^5^ cell equivalents) and immunoprecipitates were analyzed by SDS-PAGE under reducing conditions on NuPAGE 4 to 12% Bis-Tris protein gels (Thermo Fisher) and Western blotting using a Li-Cor Odyssey Sa imaging system (Li-Cor Biosciences).

### Cellular assays.

Primary CD4^+^ T cells were isolated from cones from anonymous donors with approval from the National Health Service Blood and Transplant (NHSBT) and stimulated with CD3 and CD28 MAbs on Dynabeads (Thermo Fisher) in the presence of 100 U/ml IL-2 to produce T cell blasts. A Jurkat T cell line (expressing the 1G4 TCR; provided by Oreste Acuto, University of Oxford) (33) or primary T cell blasts were transduced with lentivirus (34), and when necessary, Jurkat cells were selected for expression by using magnetic beads coated with the T12.1 CD6 MAb (ATCC). For functional assays, 96-well round-bottom plates were coated with CD3 (UCHT1; eBioscience) and/or human (T12.1) or rat (OX52; European Collection of Authenticated Cell Cultures [ECACC]) CD6 MAbs. Jurkat or activated primary CD4^+^ T cells (5 × 10^5^ cells in 200 μl) expressing CD6 or mutants were added, and the mixture was incubated for 18 h at 37°C. Cells were then stained with anti-CD69-allophycocyanin (APC) (Life Technologies) and analyzed by flow cytometry to compare MFIs of transduced cells with those of untransduced cells in each experiment for Jurkat cells and the percent positive cells in T cell blasts. T cell blasts were also analyzed for cytoplasmic IL-2 production at 18 h by blocking secretion with 10 μg/ml brefeldin A (Sigma-Aldrich). Cells were then fixed and permeabilized with fixation buffer (BD Biosciences) for 20 min at room temperature and stained with anti-IL-2–APC in Perm/wash buffer (BD Biosciences), and the percentage of positive cells was analyzed by flow cytometry.
